# Robust Estimation of Earthquake Magnitude in Indonesia Using PGD Scaling Law from Regional High-Rate GNSS Data

**DOI:** 10.3390/s25134113

**Published:** 2025-07-01

**Authors:** Thomas Hardy, Irwan Meilano, Hasanuddin Z. Abidin, Susilo, Ajat Sudrajat, Supriyanto Rohadi, Retno Agung P. Kambali, Aditya Rahman, Brilian Tatag Samapta, Muhammad Al Kautsar, Alpon Sepriando Manurung, Putu Hendra Widyadharma

**Affiliations:** 1 Geodesy and Geomatics Engineering, Faculty of Earth Science and Technology, Institut Teknologi Bandung (ITB), Bandung 40132, Indonesia; irwanm@itb.ac.id (I.M.); hzabidin@itb.ac.id (H.Z.A.); ,; 2 Indonesian Meteorology, Climatology and Geophysics Agency (BMKG), Jakarta 12710, Indonesia; supriyanto.rohadi@bmkg.go.id (S.R.); prasetyo.agung@bmkg.go.id (R.A.P.K.); rahman.aditya@bmkg.go.id (A.R.); brilian.samapta@bmkg.go.id (B.T.S.); alpon.sepriando@bmkg.go.id (A.S.M.); putu.hendra@bmkg.go.id (P.H.W.); 3 National Research and Innovation Agency (BRIN), Cibinong 16911, Indonesia; susi011@brin.go.id; 4 Indonesian Geospatial Information Agency (BIG), Cibinong 16911, Indonesia; muhammad.al@big.go.id

**Keywords:** peak ground displacement (PGD) scaling law, earthquake magnitude estimation, high-rate GNSS data, tsunami early warning

## Abstract

The accurate and timely estimation of earthquake magnitude is essential for effective tsunami early warning, particularly in seismically active regions such as Indonesia. Conventional seismic approaches are often hindered by magnitude saturation in significant events (Mw > 7.5), resulting in systematically underestimated magnitudes. To address this limitation, we develop a regional peak ground displacement (PGD) scaling law using high-rate GNSS (HR-GNSS) data from 21 moderate to large earthquakes in Indonesia. Based on 87 PGD observations, we construct a regression model that relates PGD, hypocentral distance, and moment magnitude (Mw). The PGD-derived magnitudes (MPGD) exhibit strong concordance with catalog moment magnitudes, achieving a mean absolute deviation (MAD) of 0.21 and surpassing the accuracy of previously published global models. Retrospective analyses reveal that MPGD estimates converge within 2–3 min for well-recorded events and remain robust, even for great and tsunamigenic earthquakes. These results underscore the potential of HR-GNSS data to complement conventional seismic networks, providing rapid and reliable magnitude estimates for operational tsunami early warning in Indonesia.

## 1. Introduction

Indonesia is very susceptible to earthquakes and tsunamis due to its position at the intersection of four significant tectonic plates: the Indo-Australian, Eurasian, Philippine, and Pacific plates [1,2,3,4]. This complex tectonic interaction creates a highly dynamic and seismically active environment, resulting in significant loss of life and economic devastation. For example, significant loss of life and economic loss occurred due to the 2004 Aceh earthquake and tsunami. It is estimated that the death toll reached 173,741 people, and the financial loss totaled USD 4.9 billion [5]. The Indonesian Tsunami Early Warning System (InaTEWS) was built in 2008 to provide early warning of tsunamis caused by significant offshore earthquakes, thereby lowering risks and boosting community resilience.

Earthquake magnitude estimation is crucial for earthquake and tsunami early warnings, while magnitude scaling is primarily derived from seismic sensors. However, magnitude saturation often occurs for large earthquakes (Mw > 7.5), leading to underestimated initial values. For example, in the 26 December 2004 Aceh tsunami, the Pacific Tsunami Warning Center (PTWC) initially estimated a magnitude of Mw 8.0, 11 min after the origin time (OT), 1 h later, it rose to Mw 8.5, and a few days later, this value was revised to Mw 9.2 after more seismic data became available [6,7,8]. Similarly, during the 2011 Tohoku tsunami in Japan, the Japan Meteorological Agency (JMA) first estimated a magnitude of Mw 7.9 within three minutes, later updating it to Mw 8.4, and finally to Mw 9.0 after several days [9,10]. If the actual size of the earthquakes had been determined earlier, more effective evacuations could have been possible.

Magnitude saturation occurs when seismic signals from surrounding sensors are clipped, resulting in off-scale amplitudes. Large earthquakes generate signals that surpass the seismometer’s dynamic range, leading to incorrect results [11,12,13,14]. Furthermore, acceleration recordings are utilized in the near field of major earthquakes because they do not undergo amplitude saturation. However, determining earthquake magnitude typically requires integrating acceleration records to derive displacement, a process that is often affected by baseline drift resulting from factors such as instrument tilt and rotation [13,15,16]. High-rate GNSS data, however, provide direct displacement measurements without these limitations, making them valuable for real-time earthquake magnitude estimation [13,14,15,16].

Numerous studies have demonstrated that peak ground displacement (PGD) derived from high-rate GNSS data can accurately estimate earthquake magnitude [12,13,14,15,16,17,18,19,20,21,22]. PGD is defined as the maximum displacement observed in a time series. Crowell et al. [12] introduced the PGD scaling law, which establishes a quantitative relationship between peak ground displacement and the distance from the earthquake epicentre. This algorithm enables the rapid and accurate calculation of earthquake moment magnitude (Mw) by comparing the maximum ground displacements recorded at multiple GNSS stations.

Indonesia’s Tsunami Early Warning System (InaTEWS) relies on seismometer recordings to determine earthquake parameters, including location, magnitude, and moment tensor. Despite Indonesia having 473 continuously operating GNSS stations within the Indonesian Continuously Operating Reference Station (InaCORS) network, high-rate GNSS (HR-GNSS) data has not yet been incorporated into earthquake parameter determination in InaTEWS. Instead, GNSS data is primarily used in surveying and mapping applications [23].

This paper introduces a regional PGD scaling law that utilizes PGD extracted from GNSS displacement waveforms to determine earthquake magnitude in Indonesia. Initially, we examine the attenuation relationship of PGD concerning hypocentral distance using 87 displacement records from 21 moderate to large earthquakes in Indonesia. We then use this attenuation relationship to derive a PGD scaling law. Finally, we apply the PGD scaling law to estimate and compare earthquake magnitudes with the reported moment magnitudes. The regional PGD scaling law, which was developed using Indonesia-specific GNSS data, exhibits improved accuracy over global models, as indicated by a lower mean absolute deviation (MAD), and yields robust and reliable earthquake magnitude estimates.

## 2. Materials and Methods

### 2.1. High-Rate GNSS Data Collection and Processing

**Figure 1:**
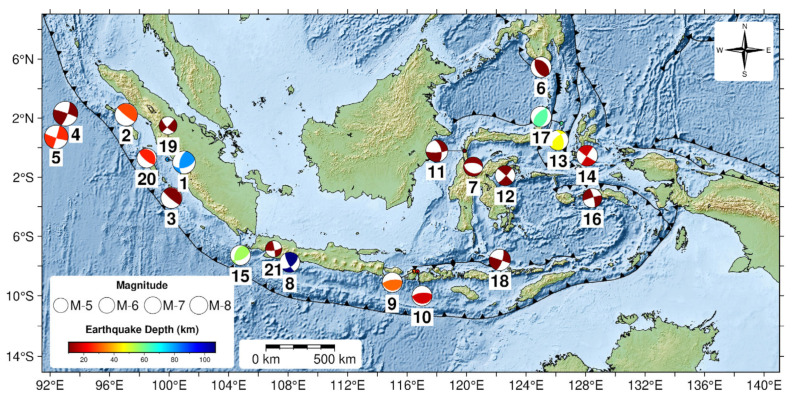
Epicenter locations for the 21 events used in this study. Colored focal mechanisms are from the Global Centroid Moment Tensor (GCMT) Catalog [26]. The circle size correlates with magnitude, while the color indicates hypocentral depth. The numbering of the focal mechanism beach balls reflects the chronological sequence of earthquake events since 2009 and correspond to the event numbering presented in Table 2.

We acquired 1 Hz high-rate GNSS data for 21 moderate to large earthquakes in Indonesia between 2009 and 2022, with magnitudes ranging from Mw 5.6 to 8.4 (Figure 1) [24]. This high-rate GNSS data was collected from two networks: the Sumatran GPS Array (SuGAr) and the Indonesia Continuously Operating Reference Station (InaCORS). SuGAr stretches over a thousand kilometres at the tectonic border where the Indo-Australian and Asian plates converge. The Earth Observatory of Singapore (EOS) manages this network, which has recently extended to include 60 continuous GNSS stations. It offers essential data for understanding the Sunda megathrust and the Sumatran fault [25]. The Geospatial Information Agency (BIG) administers InaCORS, which comprises 473 GNSS stations across Indonesia. This network may enable various applications, including high-precision mapping, navigation, and geodynamic monitoring [23].

The high-rate (HR) GNSS data for earthquake events from 2009 to 2012, obtained from the Sumatran GPS Array (SuGAr) network, consist solely of GPS observations. For earthquake events between 2017 and 2021, data from the Indonesian Continuously Operating Reference Station (InaCORS) network includes both GPS and GLONASS observations. In contrast, the HR-GNSS data for the 2022 earthquake event, also from the InaCORS network, comprises observations from GPS, GLONASS, and Galileo satellite systems. The HR-GNSS data available in the Receiver Independent Exchange Format (RINEX) mode were processed to derive kinematic displacement data using the Precise Point Positioning with Ambiguity Resolution (PPP-AR) technique [27,28,29]. We used a PRIDE PPP-AR software (version 3.0) package developed by the GNSS Research Center at Wuhan University [30]. Table 1 provides a detailed overview of the employed HR-GNSS kinematic Precise Point Positioning (PPP) processing strategies.

Ionosphere-free (IF) linear combinations were formed using GPS L1/L2, GLONASS L1/L2, and Galileo E1/E5a signals. Observations from all satellite systems were assigned equal weights. The processing utilized rapid satellite orbit and clock products, Earth Rotation Parameters (ERP), and satellite phase bias products provided by Wuhan University’s IGS Analysis Center (termed as “WUM”). Tropospheric delay was modeled using the Saastamoinen model [31] combined with the Global Mapping Function (GMF) [32]. Residual tropospheric delays were estimated as a stochastic process using a random walk model. Cycle slips and gross measurement errors were detected and corrected using the geometry-free (GF) phase combination and the Melbourne–Wübbena (MW) linear combination [33,34]. Tidal displacements were corrected in accordance with the IERS 2010 conventions [35]. Antenna phase center offsets (PCO) and phase center variations (PCV) were corrected using the absolute phase center model, IGS14.atx, provided in ANTEX format [36]. Parameter estimation was performed using a least-squares adjustment. Wide-lane integer ambiguities were resolved through a simple rounding technique, while narrow-lane ambiguities were fixed using the least-squares ambiguity decorrelation adjustment (LAMBDA) method [37].

The PPP-AR method necessitates the integration of precise satellite orbit and clock corrections, thereby ensuring centimeter-level accuracy in the displacement measurements. Due to the inherent noise levels of approximately 1–2 cm in GNSS data [15], stations farther from the earthquake hypocenter often fail to record substantial signals for certain moderate-magnitude events. Hence, we selected GNSS waveforms with peak amplitudes of at least 2 cm. We also selected HR-GNSS that have a signal-to-noise ratio (SNR) above 40 dBHz to ensure a stronger signal-to-noise level [38]. These filters retain 87 high-rate GNSS recordings in 3 components (East, North, and Up) from 21 moderate to large earthquakes in Indonesia (Table 2).

### 2.2. PGD Scaling Law

Peak ground displacement (PGD) is the maximum dynamic displacement recorded at a GNSS station during an earthquake. This value is calculated as the Euclidean norm of the three components (East (E), North (N), and Up (U)), expressed mathematically as follows:

(1) $$PGD=max\left(\sqrt{{E\left(t\right)}^{2}+{N\left(t\right)}^{2}+{U\left(t\right)}^{2}}\right)$$

PGD scaling law defines the relationship among PGD, moment magnitude (Mw), and the distance from the earthquake hypocenter to the GNSS station (R) as follows [12]:

(2) $$logPGD=A+B.Mw+C.Mw.log\left(R\right)$$

(3) $$Mw=\frac{logPGD-A}{B+C.log\left(R\right)}$$

where M_w_ is moment magnitude (M_w_) determined by the Indonesian Meteorology, Climatology, and Geophysics Agency (BMKG); R is the hypocentral distance in km; and A, B, and C are the regression coefficients.

Another study previously employed a PGD scaling law for moderate to large earthquakes, based on global HR GNSS displacement worldwide [13,14,15]. We developed the regional PGD scaling law using 87 HR Data GNSS from 21 moderate to major earthquakes in Indonesia. We applied linear regression using the least squares method to analyze the 87 PGD measurements and obtain the regression coefficients (A, B, and C). To estimate the uncertainty of these coefficients, we employed a bootstrap approach, in which 10% of the PGD data were randomly removed and the regression analysis was repeated. This procedure was performed 1000 times to estimate the variance of the coefficients, with the uncertainties reported as 95% confidence intervals.

## 3. Results

**Figure 2:**
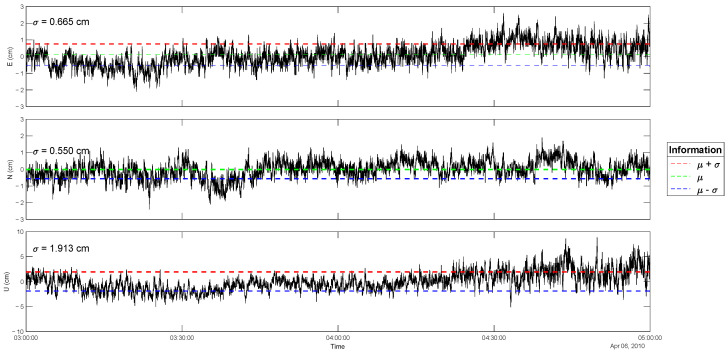
GNSS kinematic displacement with standard deviations (σ) for each component (East, North, and Up).

To evaluate the uncertainty of the GNSS kinematic displacement, we plotted the standard deviation of the three components: East (E), North (N), and Up (U). A sample of stable GNSS data was selected and recorded during a period that was unaffected by seismic shaking, several hours before the earthquake event. The GNSS data used were obtained from station PBLI, which is part of the SuGAr network, during an interval from 03:00 to 05:00 UTC, approximately 20 h before the Mw 7.7 Sinabang earthquake on 6 April 2010 (Figure 2). Based on the analysis of the standard deviation (σ) of the GNSS displacement time series, the uncertainties for the East, North, and Up components were 0.665 cm, 0.550 cm, and 1.913 cm, respectively. These values are consistent with the expected positioning accuracy using the Precise Point Positioning with Ambiguity Resolution (PPP-AR) method for hourly data, which typically yields accuracies of 0.8, 0.9, and 2.5 cm for East, North, and Up components, respectively, as published by Geng et al. (2010) [28].

**Figure 3:**
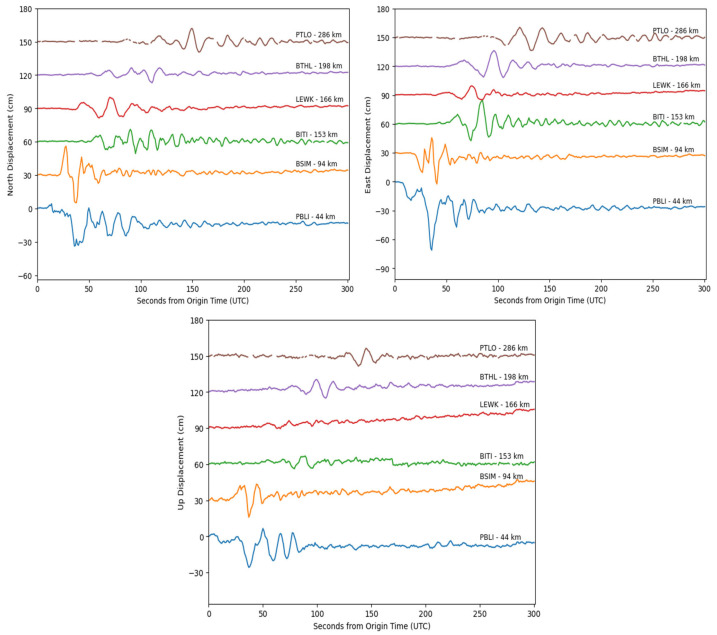
Sample displacement GNSS waveform of the Mw 7.7 Sinabang earthquake on 6 April 2010, including the East, North, and Up components. Station names and hypocentral distances are labeled.

To evaluate the effectiveness of early peak ground displacement (PGD) measurements for rapid magnitude estimation, the kinematic displacement time series was truncated to a 5–7 min window following the earthquake origin time (OT). Figure 3 displays the displacement waveforms of the Mw 7.7 Sinabang earthquake (6 April 2010) in three components (East, North, and Up), as recorded by six high-rate GNSS stations. A systematic decrease in displacement amplitude with increasing hypocentral distance is observed, which is consistent with theoretical expectations of ground motion attenuation. Figures S1–S5 in the Supplementary Materials show the epicenters and GNSS station locations alongside the displacement waveforms recorded by at least six high-rate GNSS stations for selected earthquakes. Table S1 in the Supplementary Materials presents the peak ground displacement (PGD) calculation results and the corresponding hypocentral distances for each HR GNSS station across 21 moderate to large earthquakes. The PGD values, derived from 87 HR GNSS stations, range from 2.01 to 292.96 cm. The smallest PGD (2.01 cm) was recorded at the CPMK station during the Mw 6.9 earthquake on 15 December 2017, in Tasikmalaya, West Java. In contrast, the largest PGD (292.96 cm) was observed at the PALP station for the Mw 7.5 earthquake on 28 September 2018 in Palu-Donggala, Central Sulawesi. The hypocentral distances of the HR GNSS stations ranged from 17 km to 1287 km.

**Figure 4:**
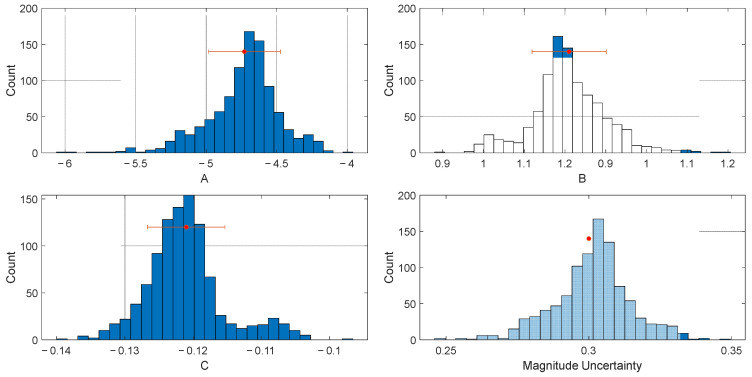
Histograms of the PGD scaling law coefficients (**A**–**C**) and the magnitude uncertainty. The red circles indicate the estimated values of the PGD scaling law coefficients, while the red error bars represent standard deviations of each coefficient.

The regression coefficients obtained from the linear regression analysis using the bootstrap method are as follows: A = −4.729 ± 0.255, B = 1.055 ± 0.045, and C = −0.121 ± 0.006, with a residual standard deviation of 0.30 magnitude units. Figure 4 depicts a histogram of the results of the bootstrap analysis for the coefficients (A, B, and C) and the magnitude uncertainty using the PGD scaling law. The peak ground displacement (PGD) scaling law was derived based on data from 87 High-Rate GNSS stations, corresponding to 21 moderate to major earthquakes in Indonesia, and is expressed as follows:

(4) $$logPGD=-4.729+1.055.Mw+-0.121.Mw\;log\left(R\right)$$

**Figure 5:**
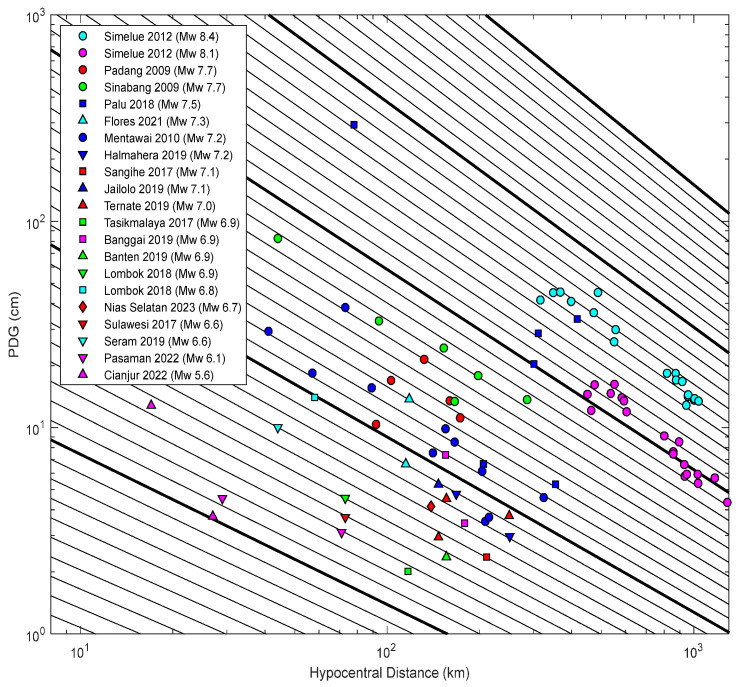
Peak ground displacement (PGD) measurements with hypocentral distance. Oblique lines are the predicted magnitudes as a function of PGD and hypocentral distance following the scaling law expressed in Equation (3).

Figure 5 illustrates the distribution of PGD as a function of hypocentral distance. PGD measurements at each station are plotted, with color and marker types distinguishing different earthquakes. The oblique lines illustrate predicted magnitudes as a function of PGD and hypocentral distance. The PGD measurements for each event generally fluctuate among the oblique lines and decrease in amplitude as the hypocentral distance increases. The plot reveals a clear attenuation trend, with PGD decreasing approximately logarithmically with distance. Higher-magnitude events consistently produce larger PGDs at all distances.

Several well-recorded large-magnitude earthquakes (e.g., the doublet Simeulue 2012, Padang 2009, Sinabang 2010, Palu-Donggala 2018) form an upper bound to the PGD distribution. Meanwhile, moderate-magnitude events (e.g., Cianjur 2022, Mw 5.6) show PGDs confined to the lower part of the graph. Additionally, shallow earthquakes, particularly strike–slip events, such as the earthquake in Palu in 2018 (Mw 7.5), generate high PGDs at relatively short hypocentral distances, highlighting the hazard potential of near-field ruptures. In contrast, deeper events (e.g., Tasikmalaya 2017, depth 107 km) generate significantly lower surface displacements, even at moderate distances.

Using the determined PGD scaling law, we compute the mean PGD magnitude (M_PGD_) estimation and its standard deviation for each of the 21 events, as detailed in Table 2. Table 2 also lists 21 earthquake events across Indonesia between 2009 and 2022 that were analyzed to evaluate the relationship between the moment magnitude (Mw) and the magnitude estimated from peak ground displacement (M_PGD_). The events exhibit diverse tectonic settings, encompassing strike–slip, reverse, and normal fault mechanisms. Most earthquakes occurred at shallow depths (<30 km), with a few exceptions, such as the Padang (81 km) and Tasikmalaya (107 km) events, which occurred at intermediate depths.

Table 2 shows the number of high-rate GNSS (HR GNSS) stations that recorded surface displacement during earthquakes, ranging from 1 to 19 per event. We conducted an extensive search for available high-rate GNSS (HR-GNSS) data associated with moderate to large earthquakes in Indonesia. However, we were able to identify only 87 HR-GNSS waveforms corresponding to 21 moderate to large events that occurred between 2009 and 2022. This limitation is primarily attributed to the availability and geographic coverage of HR-GNSS data. The Sumatran GPS Array (SuGAr), for instance, has provided high-rate data since 2009 but is geographically restricted to the Sumatran region. Additionally, the Indonesian Continuously Operating Reference Stations (InaCORS) network, which currently comprises 473 stations, began undergoing significant expansion in 2021. Despite this growth, the network remains relatively sparse, with an average inter-station spacing of approximately 72 km [39]. While this configuration is sufficient for regional-scale monitoring, it limits the spatial resolution of surface displacement measurements.

The number of GNSS stations significantly influenced the robustness of the M_PGD_ estimates. Events with more than five GNSS stations, such as the Simeulue and Mentawai earthquakes, exhibited lower standard deviation in M_PGD_. In contrast, events monitored by only one or two stations, like the Sangihe or Palu 2017 earthquakes, showed larger standard deviation ranges in magnitude estimation. Overall, M_PGD_ estimates are closely aligned with the magnitude moment (Mw), with a mean residual standard deviation of 0.3 magnitude units, indicating the robustness of PGD scaling for rapid magnitude estimation.

**Figure 6:**
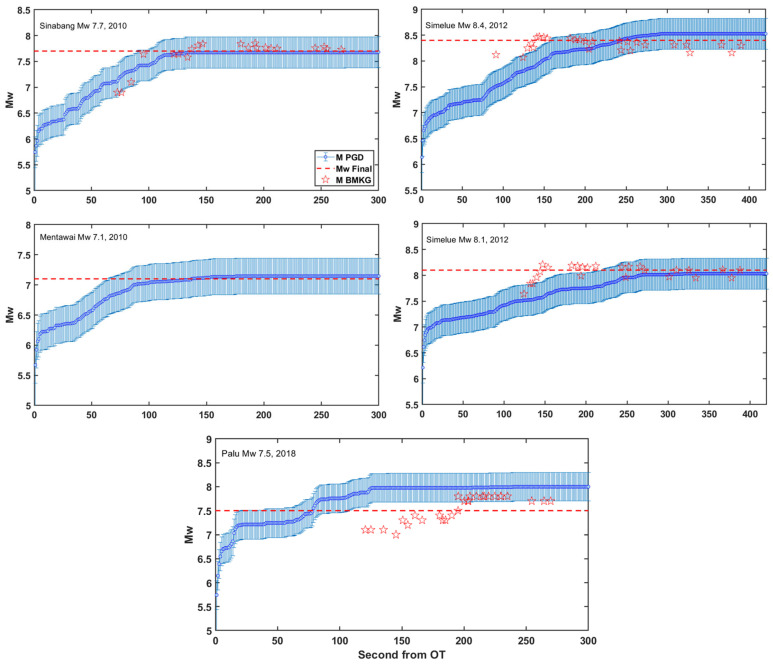
Retrospective evolution of PGD magnitude for five earthquakes, with a minimum of six GNSS stations recording displacement, utilizing the PGD scaling law (4). Magnitude calculations are shown, with error bars representing the standard error of magnitude residuals of 0.30 magnitude units. The red dashed line represents the final moment magnitude (Mw) analysis from BMKG, while the red star indicates the evolution of early seismic magnitude determination by BMKG.

We also conducted retrospective magnitude evolution estimations for several large earthquakes using high-rate GNSS data (Figure 6), focusing on events recorded by a minimum of six stations to ensure magnitude stability, with a mean error of ≤0.3 magnitude units, as found in research by Ruhl et al. [17]. The five earthquakes include the Mw 7.7 Sinabang (2010), Mw 7.2 Mentawai (2010), Mw 8.4 Simeulue (2012), Mw 8.1 Simeulue (2012), and Mw 7.5 Palu-Donggala (2018) events.

M_PGD_ estimates initially exhibit significant variability within the first 50–100 s from OT for all events due to the dynamic rupture evolution and limited early PGD observations. As more GNSS data become available and peak displacements are recorded, the magnitude estimates progressively converge toward stable values. In the case of the Sinabang (2010) and Mentawai (2010) earthquakes, the M_PGD_ stabilized within approximately 125 s from the OT, accurately matching the final Mw values of 7.7 and 7.1, respectively. Similarly, for the doublet Simeulue earthquakes (2012), the stable M_PGD_ was achieved at 8.53 ± 0.15 and 8.03 ± 0.12 within approximately 300 s from the OT, which closely correspond to the final moment magnitude from BMKG Mw 8.4 and Mw 8.1, respectively.

## 4. Discussion

Based on the peak ground displacement (PGD) magnitude estimation results for the 21 earthquakes used to develop the PGD scaling law, the standard deviation of the estimated magnitudes ranges from 0.02 to 0.74 (Table 2). Generally, lower standard deviation values are observed for earthquakes during which at least six GNSS stations recorded the displacement. For instance, significant events along the Sumatra plate boundary, such as the Mw 7.7 Sinabang earthquake (2010), the Mw 7.1 Mentawai earthquake (2010), the Mw 8.4 Simeulue earthquake (2012), and the Mw 8.1 Simeulue earthquake (2012), exhibited more minor uncertainties. This finding is consistent with the results reported by Ruhl et al. (2017) [17], which demonstrate that a minimum of six stations is necessary to achieve a mean magnitude error of less than or equal to 0.3 magnitude units. Hence, six GNSS stations measuring a static offset act as the criterion to determine the geodetic “first alert” warning in the Geodetic Alarm System (G-larmS), which is the first operational real-time geodetic system in the United States [17]. It also aligns with the findings of Gao et al. [16], who noted that an increase in the number of GNSS stations improves the accuracy of the final magnitude estimates. Notably, the estimations for the two great earthquakes in Simeulue (Mw 8.4 and Mw 8.1), with M_PGD_ values of 8.53 ± 0.15 and 8.03 ± 0.12, respectively, confirm that the PGD magnitude estimations do not exhibit saturation, even for very large events.

Regarding the evolution of M_PGD_ estimates in Figure 6, particularly for the 2012 Simeulue doublet events, the M_PGD_ values stabilize approximately 150 s later than the initial seismic magnitude estimates provided by BMKG for these two events. This apparent delay and slight uncertainty, particularly for the Mw 8.4 event (MPGD = 8.53 ± 0.15), can be attributed to the spatial configuration of the high-rate GNSS (HR-GNSS) network and the offshore locations of the earthquake hypocenters. Specifically, the Simeulue earthquakes occurred more than 300 km from the nearest HR-GNSS stations, which are situated on the Sumatran mainland (Figures S3 and S4 in the Supplementary Materials). Additionally, the spatial distribution of the GNSS stations that recorded the Simeulue doublet events was limited to a single azimuthal direction: southeast of the epicentres. Consequently, the stations required more time to capture the peak ground displacements due to the longer seismic wave travel times and the delayed arrival of surface waves at far-field GNSS stations.

In contrast, the MPGD estimates for the Sinabang Mw 7.7 and Mentawai Mw 7.1 earthquakes were obtained more rapidly (approximately 125 s after origin time) and were consistent with the final magnitudes reported by BMKG. This can be attributed to the relatively closer hypocentral distances (approximately 40–300 km) and the more azimuthally diverse spatial distribution of GNSS stations involved in these events (Figures S1 and S2 in the Supplementary Materials). These observations are consistent with the findings of Melgar et al. (2015) [13] and Ruhl et al. (2017) [17], who emphasized the importance of the density and spatial coverage of HR-GNSS networks in determining the accuracy and timeliness of M_PGD_ estimation. Despite the challenges posed by the Simeulue events, the MPGD uncertainties remained within acceptable limits (≤0.3 magnitude units), as defined by prior studies [15,17], thereby demonstrating the robustness of the PGD scaling law, even for far-field seismic events.

**Figure 7:**
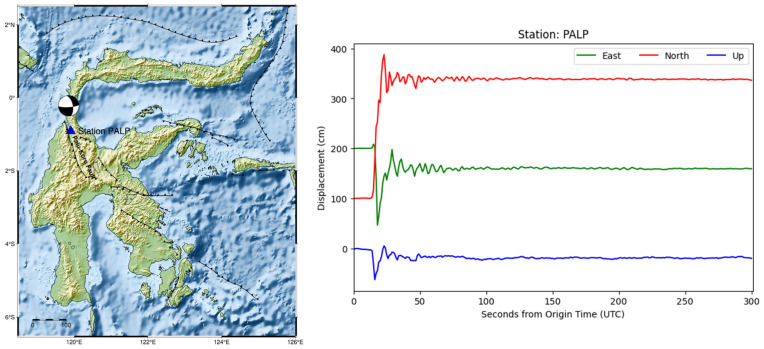
Location of the Mw 7.5 Palu-Donggala earthquake epicenter (28 September 2018) and the nearby PALP GNSS station, which recorded very large ground displacement. Displacement waveforms in the East (E), North (N), and Up (U) components are shown for the PALP station. The focal mechanism beach ball indicates the location of the earthquake epicenter.

Anomalously high PGD magnitude estimates were observed for the Mw 7.5 Palu earthquake (28 September 2018), reaching 7.98 ± 0.74. This overestimation is primarily attributed to the large peak ground displacement (2.93 m; Table S1) recorded at the PALP GNSS station, which is located approximately 78 km south of the epicenter. The rupture propagated southward along the Palu-Koro fault at supershear velocity, exceeding the crust’s shear wave speed, resulting in intense fault-parallel ground motion [40,41]. PALP, located near the fault trace, recorded a fault-parallel velocity (~1.0 m/s) substantially greater than the fault-normal component (~0.7 m/s) [42], which is consistent with theoretical and numerical models of supershear rupture dynamics [43]. The location of the epicentre and PALP station, along with the observed displacement, are presented in Figure 7. The retrospective analysis reveals that the mean M_PGD_ reached 7.5 at 78 s after OT and continued to increase, eventually stabilizing at M_PGD_ 7.98 around 126 s from OT.

Numerous studies have established peak ground displacement (PGD) scaling laws by utilizing high-rate GNSS (HR-GNSS) data from global seismic events, including those by Melgar et al. [13], Crowell et al. [14], and Ruhl et al. [15]. Building upon this foundation, the present study aims to develop a PGD scaling law based on regional HR-GNSS displacement data specifically recorded from seismic events in Indonesia. Table 3 compares the PGD scaling law coefficients derived in this study and those reported in previous works.

**Figure 8:**
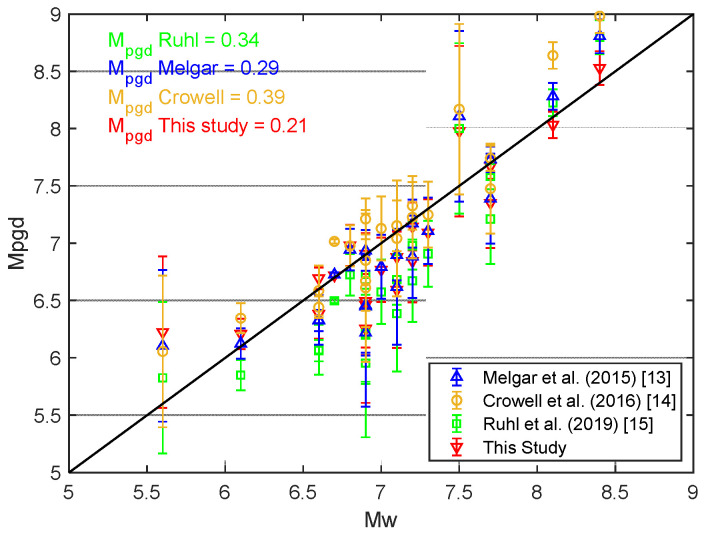
PGD magnitude (MPGD) versus the reported moment magnitude (Mw) for the 21 events. Each shape with a different color (triangle, circle, rectangle, and inverted triangle) represents a PGD scaling law, with the error bar representing the mean PGD magnitude and its standard deviation for a single event. MAD is the mean absolute deviation.

To evaluate the accuracy of the proposed PGD scaling law in estimating the magnitude from peak ground displacement (M_PGD_), a comparative analysis was conducted using 21 moderate to large earthquakes that occurred in Indonesia. The mean absolute deviation (MAD) between the MPGD-derived magnitudes and the catalog moment magnitudes (Mw) was calculated for this study, as well as for scaling laws proposed by Melgar et al. [13], Crowell et al. [14], and Ruhl et al. [15]. As illustrated in Figure 8, the MAD for the proposed model is 0.21, which is lower than the MADs of 0.29, 0.39, and 0.34 for the models by Melgar et al., Crowell et al., and Ruhl et al., respectively.

Based on Indonesia-specific high-rate GNSS data, the regional PGD scaling law developed in this study demonstrates improved accuracy over global models, as reflected by a lower mean absolute deviation (MAD), and yields robust and reliable earthquake magnitude estimates. These results indicate that the PGD scaling law derived from this study yields a more accurate fit, capturing the regional tectonic characteristics of the Indonesian region.

We acknowledge that the present study does not include cross-validation with alternative GNSS-based magnitude estimation techniques, nor does it incorporate independent datasets for external verification. We fully agree that future studies should incorporate cross-validation using established GNSS-based magnitude estimation approaches (e.g., Peak Ground Velocity (PGV) [44], W-phase inversion [45], or seismogeodetic [46] methods) and should leverage independent regional and global datasets. Such efforts will be crucial to further validating the generalizability and operational robustness of the proposed PGD scaling law.

While our regional HR-GNSS dataset offers a valuable foundation, we acknowledge that certain events were captured by only a limited number of GNSS stations, which may impact the robustness and precision of the corresponding magnitude estimates. Future research should incorporate a greater number of HR-GNSS stations as the national GNSS network (InaCORS) infrastructure continues to expand. Additionally, the integration of global datasets will be pursued to further strengthen the empirical foundation and improve the statistical reliability of the proposed PGD scaling model.

We also conducted a magnitude estimation evaluation of the proposed regional PGD scaling law data for two earthquakes outside Indonesia: the 2017 Tehuantepec Mw 8.2 earthquake in Mexico (using 7 HR-GNSS stations) and the 2016 Kaikoura Mw 7.8 earthquake in New Zealand (using 36 HR-GNSS stations). High-rate GNSS data for both earthquakes were obtained from the Supplementary Materials of Ruhl et al. (2019) [15]. The results are presented in Table 4, which demonstrates the reliability of the proposed regional PGD scaling law in providing robust earthquake magnitude estimations for events in other regions with different tectonic settings, with a standard deviation of less than 0.3 magnitude units.

Based on the standard operating procedure of the Indonesia Tsunami Early Warning System (InaTEWS), since 2024, real-time earthquake parameters such as origin time, hypocenter location, and magnitude must be provided by BMKG in less than 3 min [47]. Based on the results of the retrospective analysis of several large earthquakes, the magnitude estimation results were obtained using stable GNSS high-rate PGD data in less than 3 min for events like the Sinabang earthquake, Mentawai earthquake, and Palu-Donggala earthquake. However, for the Simeleu doublet earthquake, where the hypocenter location in the ocean is quite far from the Sumatra mainland where the GNSS sensor is located (around 300 km), a stable magnitude estimate can be obtained approximately 300 s after OT. The results reaffirm the capability of GNSS displacement data to complement and enhance traditional seismic networks to provide reliable earthquake magnitude estimates that are crucial in tsunami and earthquake early warning systems, particularly for large and tsunamigenic events in the Indonesian region. This demonstrates that PGD magnitude has excellent potential for real-time implementation in the Indonesian Tsunami Early Warning System.

For future operational implementation, it is essential to address critical factors such as station density and data transmission latency, as these will significantly affect the speed and reliability of real-time magnitude estimation. Overcoming these challenges is crucial for the effective integration of PGD-based magnitude estimation into tsunami and earthquake early warning systems. Geng et al. (2013) [46] reported that under operational conditions, the transmission of high-rate GNSS data from observation stations to a central processing server typically experiences a latency of approximately 0.4 to 1.0 s. Satellite clock corrections and fractional cycle bias (FCB) parameters are continuously estimated and promptly disseminated to client processors. Thereafter, Precise Point Positioning with Ambiguity Resolution (PPP-AR) can be performed at individual stations on an epoch-by-epoch basis, with an additional processing delay of approximately 1 s following data reception at the analysis center. Thus, in accordance with retrospective analyses of several large earthquakes with well-recorded displacements, where magnitude estimates were obtained within 2–3 min, and considering a data latency of approximately 2 s, it is feasible to achieve stable and reliable magnitude estimates that are suitable for use by tsunami or earthquake early warning centers, especially when using near-field GNSS stations.

Compared to traditional magnitude estimation methods (e.g., Gutenberg, 1945 [48]), which are based on seismic wave amplitudes, the PGD-based approach offers key advantages, particularly for large and tsunamigenic earthquakes. Traditional methods are susceptible to magnitude saturation, wherein recorded amplitudes no longer scale with earthquake size, leading to underestimation in significant events. In contrast, PGD derived from high-rate GNSS measurements captures the permanent ground displacement, making it more robust for characterizing large ruptures. Moreover, PGD estimates are less sensitive to path and source effects and provide stable magnitude estimates within minutes of the event. These features make the PGD approach especially valuable for tsunami early warning systems. However, we acknowledge that the PGD-Mw relationship is empirical and may vary by region. Therefore, while promising, the universality of this method requires further validation through regional calibration and cross-comparison with other geodetic techniques.

## 5. Conclusions

We propose a regional peak ground displacement (PGD) scaling law for rapid and reliable earthquake magnitude estimation in Indonesia. This model is developed using 87 high-rate GNSS (HR-GNSS) displacement records from 21 moderate to large earthquakes and establishes a robust empirical relationship among PGD, hypocentral distance, and moment magnitude (Mw). The resulting PGD-derived magnitudes (MPGD) demonstrate strong agreement with reported catalog Mw values, achieving a mean absolute deviation (MAD) of 0.21 and outperforming previously published global models.

The proposed regional PGD scaling law has also been evaluated using data from two earthquakes outside the Indonesian region, involving 42 high-rate GNSS stations. The standard deviation of the estimated magnitudes was found to be less than 0.3 magnitude units, indicating that the regional PGD scaling law is also reliable for earthquake magnitude estimation in other regions with different tectonic settings.

Notably, the proposed scaling law shows no evidence of magnitude saturation, even for great earthquakes (Mw > 8.0), and maintains accuracy across various tectonic regimes, including strike–slip, reverse, and normal fault mechanisms. Retrospective analyses further confirm that for events recorded by six or more GNSS stations, PGD-based magnitude estimates converge rapidly, typically within 2–3 min of the origin time, which is in accordance with the operational requirements of the Indonesia Tsunami Early Warning System (InaTEWS). These findings reaffirm the value of HR-GNSS displacement data as a complementary tool for seismic networks for real-time magnitude estimation, particularly in tectonically complex and tsunami-prone regions like Indonesia.

We acknowledge the importance of increasing the number of GNSS stations and expanding the sample size of earthquake events to enhance the robustness and generalizability of the PGD scaling law. Future studies should prioritize the incorporation of additional HR-GNSS recordings from a broader range of seismic events, both regionally and globally, to strengthen the empirical foundation and improve the statistical reliability of the model.

For continuous improvement, further validation using real-time HR GNSS displacement data from future seismic events in Indonesia will be essential to evaluate the scalability and operational reliability of the proposed model. However, challenges such as station density and data transmission latency remain key considerations for future works on real-time operational implementation.

## Figures and Tables

**Table 1:** HR-GNSS kinematic PPP processing strategies.

Item	Strategies
PPP mode	Ionospheric-free (IF) combination PPP
Observation	GPS/GLONASS/Galileo pseudorange and phase observation
Sampling rate	1 Hz
Signal frequency	GPS(LI/L2), GLONASS(L1/L2), Galileo(E1/E5a)
Satellite orbit, clock, and ERP	WUM Rapid product
Cutoff elevation	7°
Estimation method	Least-squares principle of the parameter elimination–recovery method
Coordinate reference system	IGS14
Antenna phase center correction	Corrected using IGS14.atx in ANTEX format
Tropospheric delay	Corrected by the Saastamoinen model; combined with GMF projection
Ionospheric delay	Eliminated by the IF combination
Tide correction	IERS 2010 convention
Cycle slip detection	GF + MW
Ambiguity resolution	LAMBDA for narrow lane and rounding for wide lane

**Table 2:** Earthquake information and computed mean peak ground displacement (PGD) magnitudes for 21 moderate to large events used in this study. Earthquake parameters (origin time, hypocenter, and Mw) were obtained from the Indonesian Meteorology, Climatology, and Geophysics Agency (BMKG). The source mechanism was analyzed using Global Centroid Moment Tensor (GCMT) data [26].

No	Event Name	Origin Time (UTC)	Longitude (°)	Latitude (°)	Depth (km)	Mechanism	M_w_	M_PGD_	Number of GNSS Station
1	Padang, West Sumatra	30 September 2009 10:16:10	99.88	−0.80	81	Reverse	7.6	7.35 ± 0.39	5
2	Sinabang, North Sumatra	6 April 2010 22:15:03	97.11	2.24	29	Thrust	7.7	7.68 ± 0.11	6
3	Mentawai, West Sumatra	25 October 2010 14:42:20	100.16	−3.41	10	Reverse	7.1	7.15 ± 0.21	11
4	Simelue, Aceh	11 April 2012 08:38:34	93.03	2.31	10	Strike slip	8.4	8.53 ± 0.15	18
5	Simelue, Aceh	11 April 2012 10:43:11	92.41	0.73	26	Strike slip	8.1	8.03 ± 0.12	19
6	Sangihe, North Sulawesi	28 April 2017 20:23:18	125.00	5.45	10	Reverse	7.1	6.59 ± 0.51	1
7	Palu, Central Sulawesi	29 May 2017 14:35:23	120.44	−1.29	11	Normal	6.6	6.38 ± 0.22	1
8	Tasikmalaya, West Java	15 December 2017 16:47:58	108.11	−7.75	107	Strike slip	7.2	6.25 ± 0.65	1
9	Lombok, West Nusa Tenggara	5 August 2018 11:46:37	116.47	−8.35	32	Reverse	6.8	6.98 ± 0.18	1
10	Lombok, West Nusa Tenggara	19 August 2018 14:56:27	116.7	−8.37	18	Reverse	6.9	6.50 ± 0.40	1
11	Palu-Donggala, Central Sulawesi	28 September 2018 10:02:44	119.85	−0.22	10	Strike slip	7.5	7.98 ± 0.74	6
12	Banggai Island, Central Sulawesi	12 April 2019 11:40:50	122.59	−1.89	23	Strike slip	6.8	6.91 ± 0.18	2
13	Ternate, North Maluku	7 July 2019 15:08:42	126.16	0.51	47	Reverse	7.0	6.77 ± 0.28	3
14	Halmahera, North Maluku	14 July-2019 09:10:51	128.1	−0.54	17	Strike slip	7.1	6.84 ± 0.36	2
15	Sumur, Banten	2 August 2019 12:03:27	104.79	−7.27	55	Reverse	6.9	6.46 ± 0.44	1
16	Ambon, Maluku	25 September -2019 23:46:45	128.45	−3.42	10	Strike slip	6.6	6.69 ± 0.09	1
17	Jailolo, North Maluku	14 November 2019 16:17:43	126.37	1.66	62	Reverse	7.1	6.88 ± 0.22	1
18	Flores, East Nusa Tenggara	14 December 2021 03:20:23	122.23	−7.59	10	Strike slip	7.3	7.09 ± 0.29	2
19	Pasaman, West Sumatra	25 February 2022 01:39:29	99.93	0.14	10	Strike slip	6.1	6.21 ± 0.13	2
20	Nias Selatan, West Sumatra	13 March 2022 21:09:22	98.5	−0.71	25	Reverse	6.7	6.72 ± 0.02	1
21	Cianjur, West Java	21 November 2022 06:21:10	107.03	−6.85	11	Strike slip	5.6	6.22 ± 0.66	2

**Table 3:** Comparison of several PGD scaling law coefficients.

A	B	C	HR GNSS Data	Origin
−4.434	1.047	−0.138	Global, 10 earthquakes, 1321 HR GNSS Data	Melgar et al. (2015) [13]
−6.687	1.500	−0.214	Global, 3 earthquakes, 112 HR GNSS Data	Crowell et al. (2016) [14]
−5.919	1.009	−0.145	Global, 29 earthquakes, 3433 HR GNSS Data	Ruhl et al. (2019) [15]
−4.729	1.005	−0.121	Regional (Indonesia), 21 earthquakes, 87 HR GNSS Data	This study

**Table 4:** Evaluation of the proposed regional PGD scaling law using data from 2 earthquakes and 42 HR-GNSS stations outside Indonesia, compared with existing global PGD scaling laws.

No	Earthquake	PGD Scaling Law (M_PGD_ ± Std.Dev)			
		Melgar (2015) [13]	Crowell (2016) [14]	Ruhl (2019) [15]	This Study
1	Tehuepec Mw 8.2 2017, Mexico	8.38 ± 0.35	8.60 ± 0.49	8.32 ± 0.36	8.13 ± 0.28
2	Kaikoura Mw 7.8, 2016, New Zealand	7.91 ± 0.21	8.06 ± 0.28	7.80 ± 0.20	7.77 ± 0.15

## Data Availability

Earthquake parameters (origin time, hypocenter, and Mw) were obtained from the InaTEWS Earthquake Repository, which was developed by the Meteorological, Climatological, and Geophysical Agency of Indonesia (BMKG, https://repogempa.bmkg.go.id/, accessed on 12 November 2024). The source mechanism was obtained from the Global Centroid Moment Tensor (GCMT) catalog (https://www.globalcmt.org/CMTsearch.html, accessed on 12 November 2024).

## Supplementary Materials

The following supporting information can be downloaded at https://www.mdpi.com/article/10.3390/s25134113/s1: Figure S1: Epicenter, GNSS station location and displacement waveform in 3 components (E, N, and U) of Mw7.7 Sinabang, 6 April 2010; Figure S2: Epicenter, GNSS station location and displacement waveform in 3 components (E, N, and U) of Mw7.1, Mentawai, 25 October 2010; Figure S3: Epicenter, GNSS station location, and displacement waveform in 3 components (E, N, and U) of Mw8.4, Simelue, 11 April 2012; Figure S4: Epicenter, GNSS station location, and displacement waveform in 3 components (E, N, and U) of Mw8.1, Simelue, 11 April 2012; Figure S5: Epicenter, GNSS station location, and displacement waveform in 3 components (E, N, and U) of Mw7.5, Palu-Donggala, 28 September 2018; Table S1: All peak ground displacement (PGD) values and hypocentral displacement in each stations for 21 moderate and large earthquakes.
