# Supplementary material for: Robust Estimation of Earthquake Magnitude in Indonesia Using PGD Scaling Law from Regional High-Rate GNSS Data
Source: Sensors (Basel). 2025 Jul 1;25(13):4113. doi: 10.3390/s25134113 (PMC12252110; doi:10.3390/s25134113)
Supplement: Supplementary file 1 [file sensors-25-04113-s001.zip › sensors-3660350-supplementary.pdf]

Supplementary materials to

Robust Estimation of Earthquake Magnitude in Indonesia Using PGD Scaling Law from Local High-Rate GNSS Data

Introduction:

This supplemental contains five figures (Figures S1-S5) and one table (Table S1)

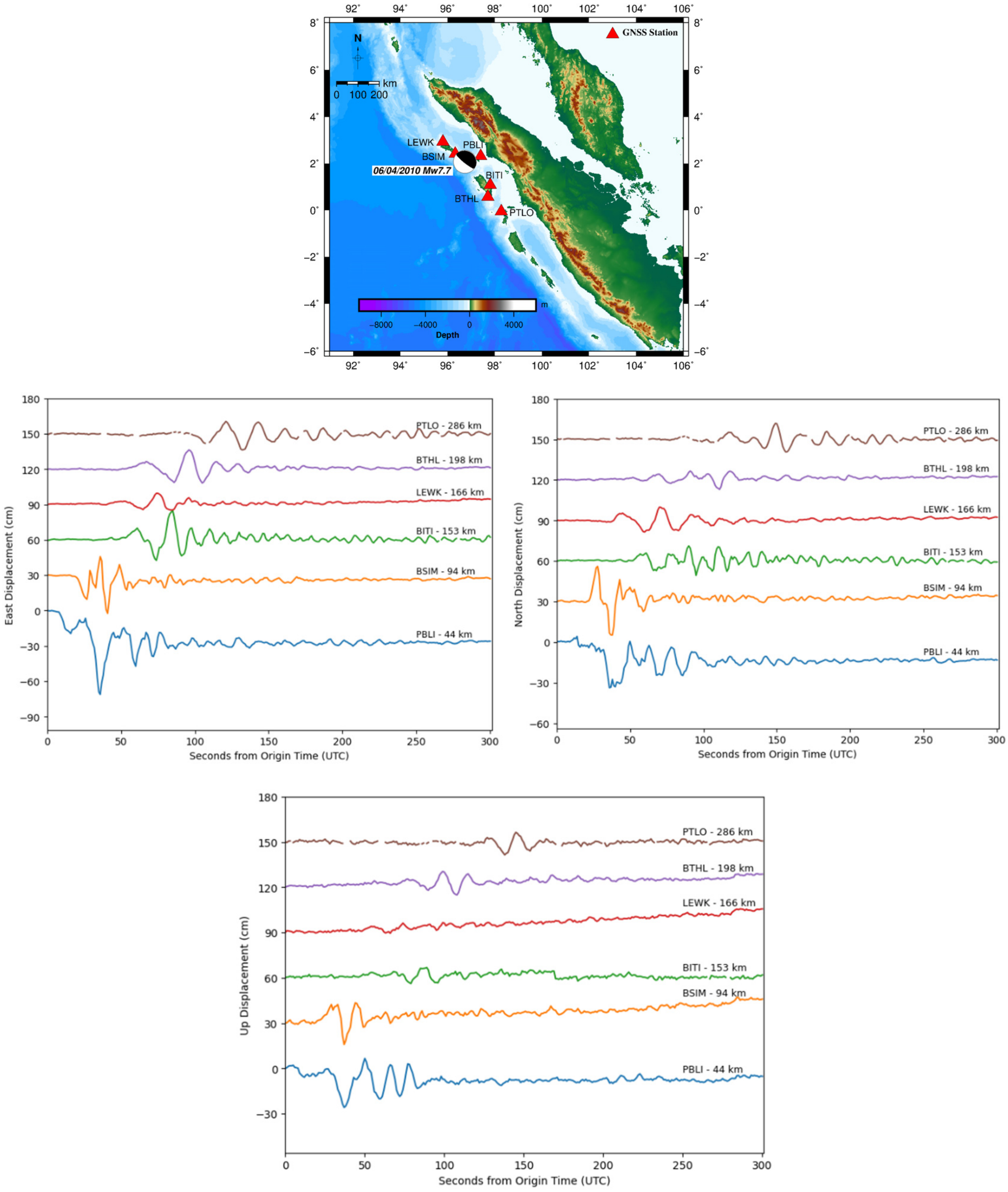

Figure S1. Epicenter, GNSS Station location and displacement waveform in 3 components (E, N, U) of Mw7.7 Sinabang, 6th April 2010

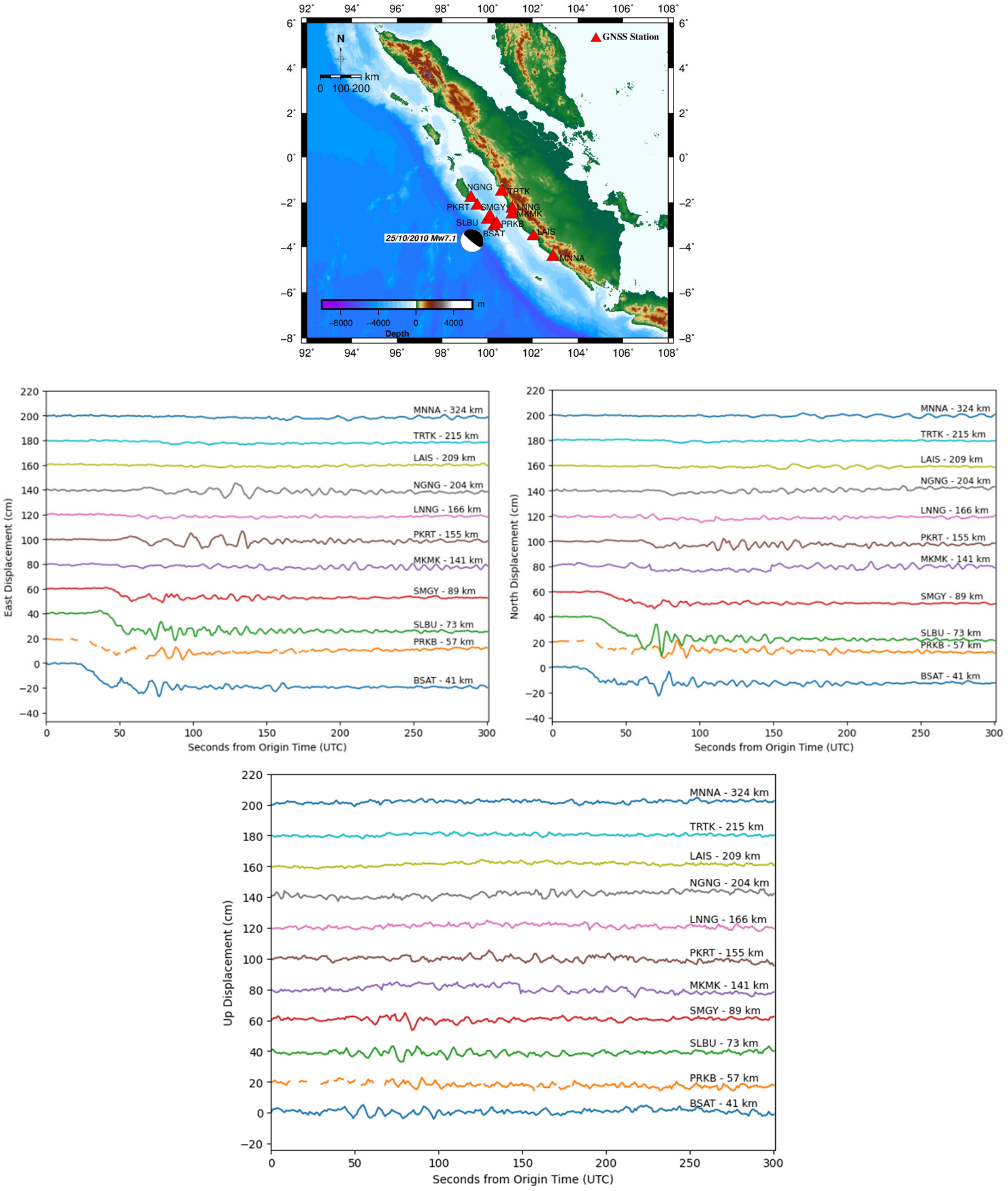

Figure S2. Epicenter, GNSS Station location and displacement waveform in 3 components (E, N, U) of Mw7.1, Mentawai, 25th October 2010

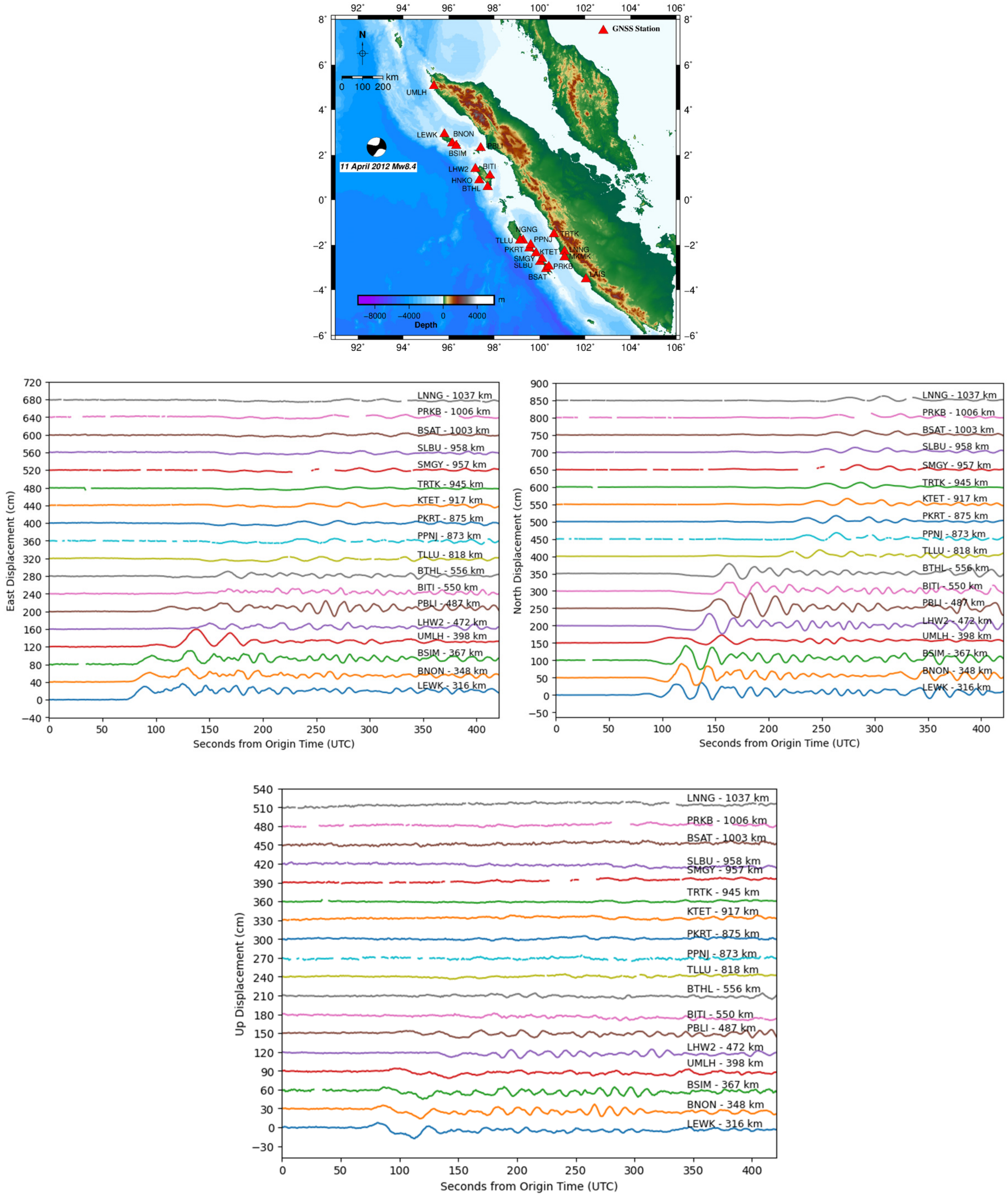

Figure S3. Epicenter, GNSS Station location, and displacement waveform in 3 components (E, N, U) of Mw8.4, Simelue, 11th April 2012.

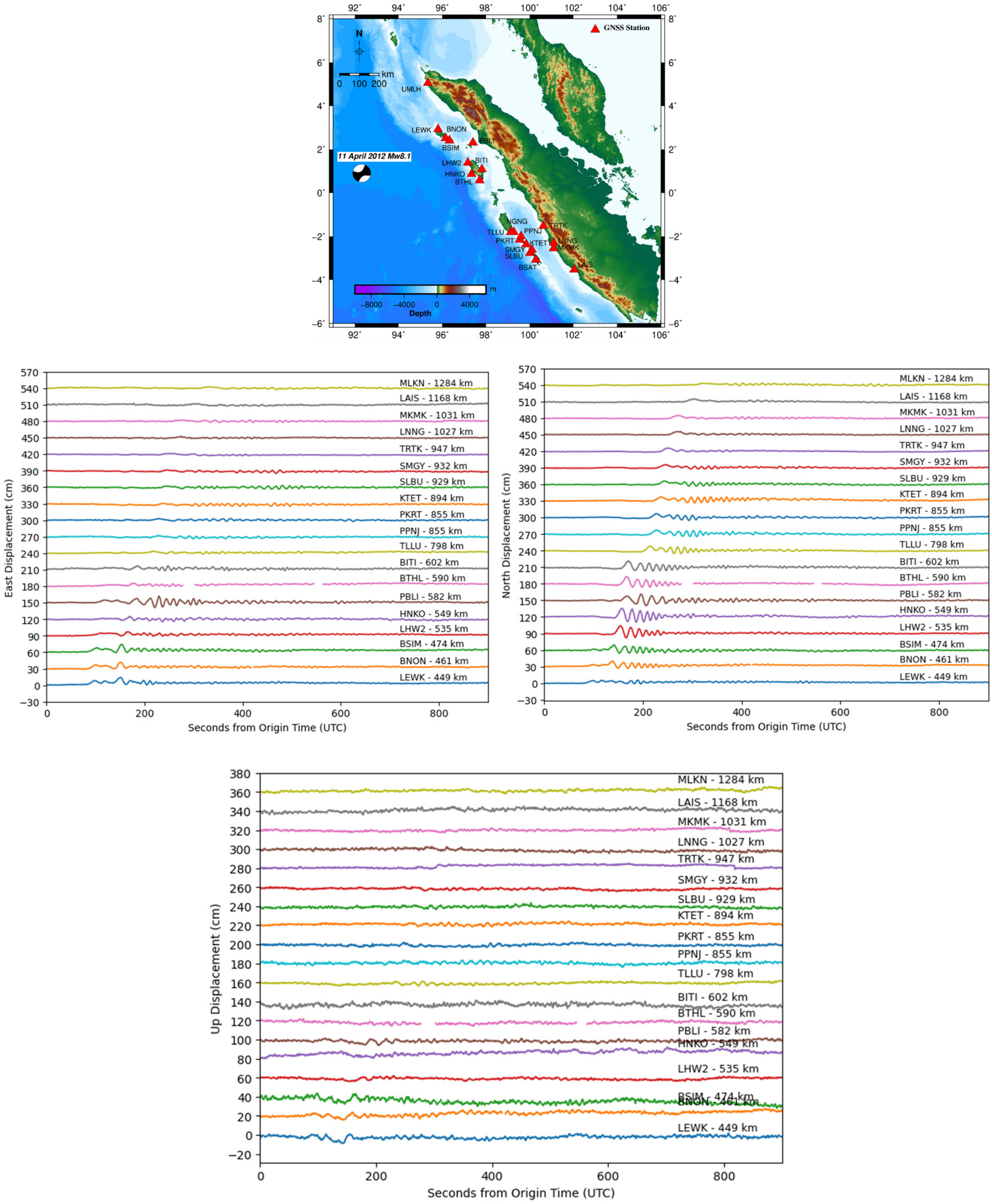

Figure S4. Epicenter, GNSS Station location, and displacement waveform in 3 components (E, N, U) of Mw8.1, Simelue, 11th April 2012.

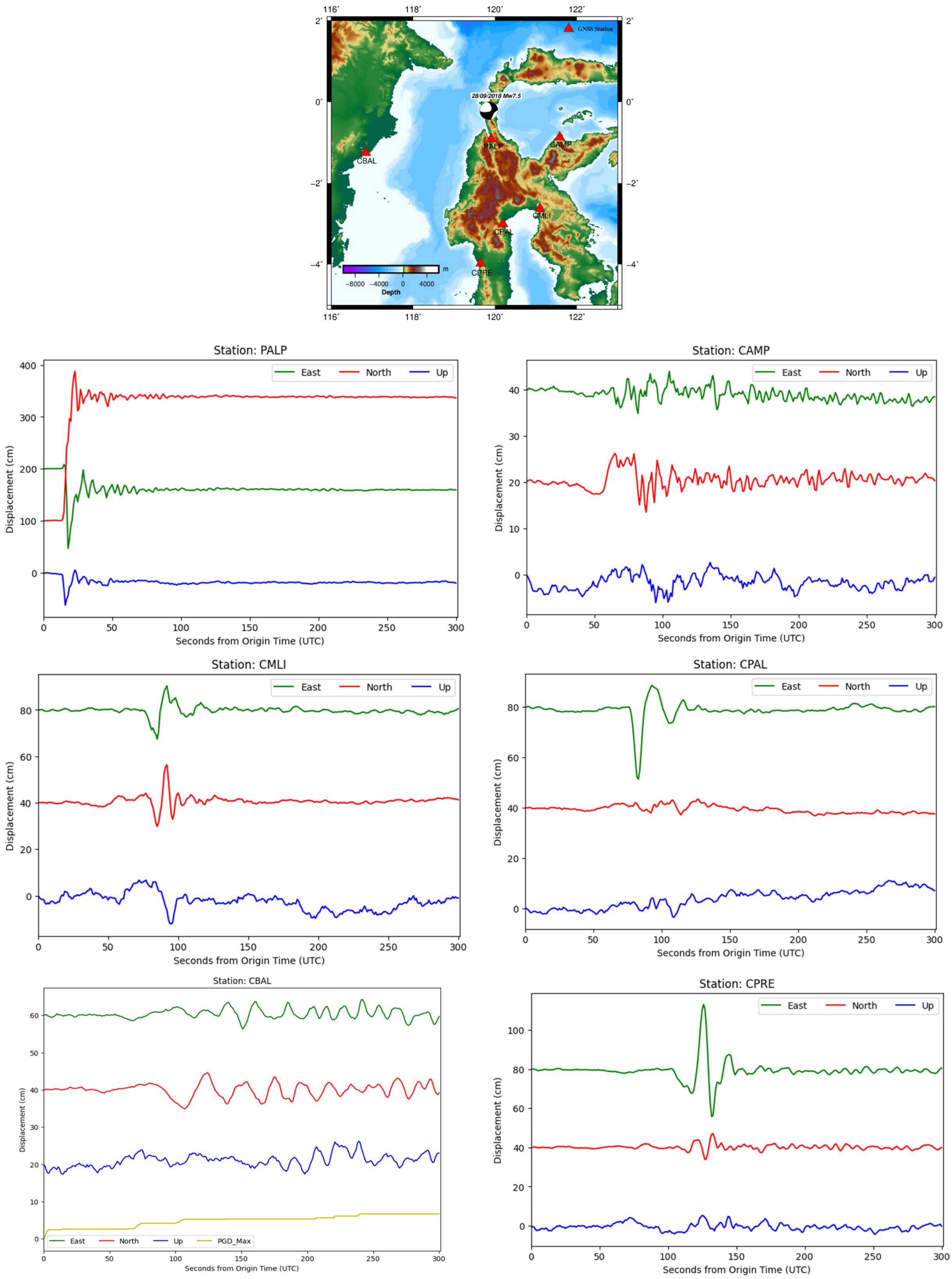

Figure S5. Epicenter, GNSS Station location, and displacement waveform in 3 components (E, N, U) of Mw7.5, Palu-Donggala, 28th September 2018.

Table S1. All Peak Ground Displacement (PGD) value and hypocentral displacement in each stations for 21 moderate and large earthquakes

No	Event	Mw	GNSS Station	PGD (cm)	Hypocentral distance (km)
1	Mw 7.7, 30 September 2009, Padang, Sumatera Barat	7.7	TIKU	10.37	92
			PSKI	16.93	103
			MSAI	21.41	132
			TLLU	13.5	160
			PKRT	11.15	173
2	Mw 7.7, 6 April 2010, Sinabang, Sumatera Utara	7.7	PBLI	82.55	44
			BSIM	32.89	94
			BITI	24.31	153
			LEWK	13.39	166
			BTHL	17.86	198
			PTLO	13.66	286
3	Mw 7.2, 25 Oktober 2010, Mentawai, Sumatera Barat	7.2	BSAT	29.29	41
			PRKB	18.37	57
			SLBU	38.21	73
			SMGY	15.58	89
			MKMK	7.54	141
			PKRT	9.87	155
			NGNG	8.51	166
			LNNG	6.14	204
			LAIS	3.51	209
			TRTK	3.68	215
			MNNA	4.58	324
4	Mw 8.4, 11 April 2012, Simelue, Aceh	8.4	LEWK	41.52	316
			BNON	45.06	348
			BSIM	45.37	367
			UMLH	40.87	398
			LHW2	36.05	472
			PBLI	45.18	487
			BITI	26.07	550
			BTHL	29.82	556
			TLLU	18.33	818
			PPNJ	18.31	873
			PKRT	17.01	875
			KTET	16.72	917
			TRTK	12.84	945
			SMGY	14.17	957
			SLBU	14.42	958
			BSAT	13.6	1003
			PRKB	13.81	1006
			LNNG	13.44	1037
5	Mw 8.1, 11 April 2012, Simelue, Aceh	8.1	LEWK	14.51	450
			BNON	12.12	463
			BSIM	16.13	475

			LHW2	14.63	536
			HNKO	16.22	551
			PBLI	13.95	582
			BTHL	13.47	592
			BITI	11.93	604
			TLLU	9.12	801
			PPNJ	7.64	857
			PKRT	7.45	857
			KTET	8.53	896
			SLBU	6.62	931
			SMGY	5.81	934
			TRTK	5.94	949
			LNNG	5.94	1030
			MKMK	5.37	1033
			LAIS	5.70	1171
			MLKN	4.34	1287
6	Mw 7.1, 28 April 2017, Kepulauan Sangihe, Sulawesi Utara	7.1	CTHN	2.36	211
7	Mw 6.6, 29 Mei 2017, Sulawesi, Indonesia	6.6	PALP	3.68	73
8	Mw 6.9, 15 Desember 2017, Tasikmalaya, Jawa Barat	7.2	CPMK	2.01	117
9	Mw 6.8, 5 Agustus 2018, Lombok, NTB	6.8	CMAT	14	58
10	Mw 6.9, 19 Agustus 2018, Lombok, NTB	6.9	CMAT	4.56	73
11	Mw 7.5, 28 September 2018, Donggala, Sulawesi Tengah	7.5	PALP	292.96	78
			CAMP	6.67	206
			CMLI	20.32	301
			CPAL	28.61	311
			CBAL	5.31	354
			CPRE	33.58	417
12	Mw 6.9, 12 April 2019, Banggai Kepulauan, Sulawesi Tengah	6.9	CAMP	7.38	155
			CMLI	3.44	179
13	Mw 7.0, 7 Juli 2019, Ternate, Maluku Utara	7.0	CTER	2.94	147
			CBIT	4.52	156
			CTBL	3.73	250
14	Mw 7.2, 14 Juli 2019, Halmahera, Maluku Utara	7.2	CTER	4.77	168
			CTBL	2.97	251
15	Mw 6.9, 2 Agustus 2019, Sumur, Banten	6.9	CMLP	2.36	156
16	Mw 6.6, 25 September 2019, Ambon, Maluku	6.6	CAMB	10.03	44
17	Mw 7.1, 14 November 2019, Jailolo, Maluku Utara	7.1	CBIT	5.3	147
18	Mw 7.3, 14 Desember 2021, Flores, NTT	7.3	CMRE	6.66	115

			CUKA	13.75	118
19	Mw 6.1, 25 Februari 2022, Pasaman, Sumatera Barat	6.1	CPSM	4.54	29
			CBKT	3.11	71
20	Mw 5.6, 21 November 2022, Cianjur, Jawa Barat	5.6	CJUR	12.81	17
			CLDO	3.71	27
21	Mw 6.7, 13 Maret 2023, Nias Selatan, Sumatera Barat	6.7	CTEK	4.15	139
